# Measuring and adapting to climate change in HIV programmes

**DOI:** 10.1016/S2352-3018(24)00231-5

**Published:** 2024-10-08

**Authors:** Nathan Ford, Anne Hammill

**Affiliations:** 1 Global HIV, Hepatitis and STIs Programs, World Health Organization, Geneva, Switzerland; 2 Centre for Infectious Disease Epidemiology & Research, School of Public Health, University of Cape Town, Cape Town, South Africa; 3 International Institute for Sustainable Development, Geneva, Switzerland

The past 9 years were the nine warmest years on record. Worldwide, extreme weather events are becoming increasingly common because of climate change and have severe socioeconomic effects, including on health. According to a NASA analysis of global daily temperature data, 2023 was by far the warmest year on record and 2024 is likely to be warmer still.

As global temperatures increase, so do the frequency and intensity of extreme weather events and the scale and severity of the socioeconomic effects they inflict on communities around the world. As stated in *The 2023 report of the* Lancet *Countdown on health and climate change: the imperative for a health-centred response in a world facing irreversible harms*,^1^ economic losses from extreme weather increased between 2010–14 and 2018–22, amounting to US$264 billion in 2022 alone, with heat exposure leading to global potential income losses worth $863 billion. Adults older than 65 years and infants younger than 1 year are now exposed to twice as many heatwave days as during 1986–2005 and heat-related deaths of people older than 65 years have increased by 85% since 1990–2000, with substantial increases projected, underscoring the urgency of climate change adaptation and mitigation efforts.^1^

A growing number of studies have described the negative impact of extreme weather events and slow-onset changes on multiple health outcomes, including increases in the risk of vector-borne, foodborne, and waterborne diseases; adverse cardiovascular and respiratory outcomes; poor birth outcomes; and diverse mental health problems. Extreme weather events have direct effects (eg, drowning and injuries) and indirect effects (eg, crop failures leading to malnutrition, environmental toxin exposure, and armed conflict) on health (figure).^2^ Displacement and involuntary migration as a result of extreme weather events lead to increased vulnerability and poor health outcomes: according to the World Bank, up to 216 million people could become internal climate migrants by 2050.^3^

A systematic review by Collins C Iwuji and colleagues^4^ in *The Lancet HIV*, including studies published up to Aug 31, 2023, summarises what is known about the relationship between extreme weather events and HIV. The 27 included studies reported negative effects of extreme weather events, including increased risk behaviour, reduced HIV testing, delays in treatment initiation, treatment interruption, and reduced CD4 cell count leading to advanced HIV disease. The review adds to evidence from a modelling study,^5^ highlighted in *The Lancet HIV* earlier this year, which suggests an increase of up to 16 million HIV cases by 2050 as a result of rising temperatures and no reduction in carbon emissions.^5^

Studies published in the year since the review coverage ended reinforce these findings. Two recent studies stand out. A study of 270 708 people with HIV across six southern African countries with high burdens of HIV found that lower rainfall than usual was associated with higher mortality and unsuppressed viral loads, which can be attributed to changing behaviours and food and income insecurity.^6^ A second study, using data from population surveys (102 081 respondents) from five southern African countries, found an association between drought, poverty, transactional sex, and recent HIV infection.^7^ Evidence linking climate change and negative HIV outcomes will continue to accumulate. A more comprehensive assessment of the impacts of climate change on health will require up-to-date global datasets, as have been developed to monitor the impact of climate change on other crucial areas such as crop production.^8^

Many factors can explain the effect of extreme weather on HIV outcomes: population displacement, financial stressors, increases in physical and sexual violence, and disrupted access to health care. Collins and colleagues^4^ used systems thinking to organise their findings into five themes: economic and livelihood conditions; psychosocial factors; infrastructure damage and operational challenges; migration and displacement; and associated medical conditions and health-care needs.

Assessing the effects of climate change on HIV outcomes is important, but how should the health system adapt? It has long been established that when health systems are overwhelmed from major shocks and people do not access needed care, incidence of and mortality from preventable and treatable conditions increases. In the early months of the COVID-19 pandemic, WHO published guidance outlining key operational strategies for maintaining essential health services during a crisis. For HIV, these strategies included multi-month dispensing of antiretrovirals and other essential medicines, prioritised HIV testing for individuals at high risk and scaled-up provision of self-testing, and electronic and mobile health strategies for counselling and adherence support;^9^ many of these approaches have since been adopted as part of routine care. HIV programmes should integrate climate considerations into HIV policies, including climate-informed health early warning systems to address risk behaviour. More recent considerations for health service can be found in WHO’s operational framework for building climate-resilient and low-carbon health systems.^10^

Crises can accelerate the introduction of beneficial interventions and highlight the investments needed to make health systems more resilient to a range of shocks and stresses—including extreme weather events. More research will help to improve our understanding of the links between climate change and HIV and to identify effective interventions. However, investment in building resilience cannot be the only line of defence in securing healthier and more prosperous outcomes—especially among the world’s most vulnerable communities. The world must also act quickly to reduce harmful emissions and limit global warming to avoid the worst effects of climate change. The health sector, which is responsible for up to 5% of global carbon emissions, has a crucial part to play.

## Figures and Tables

**Figure:**
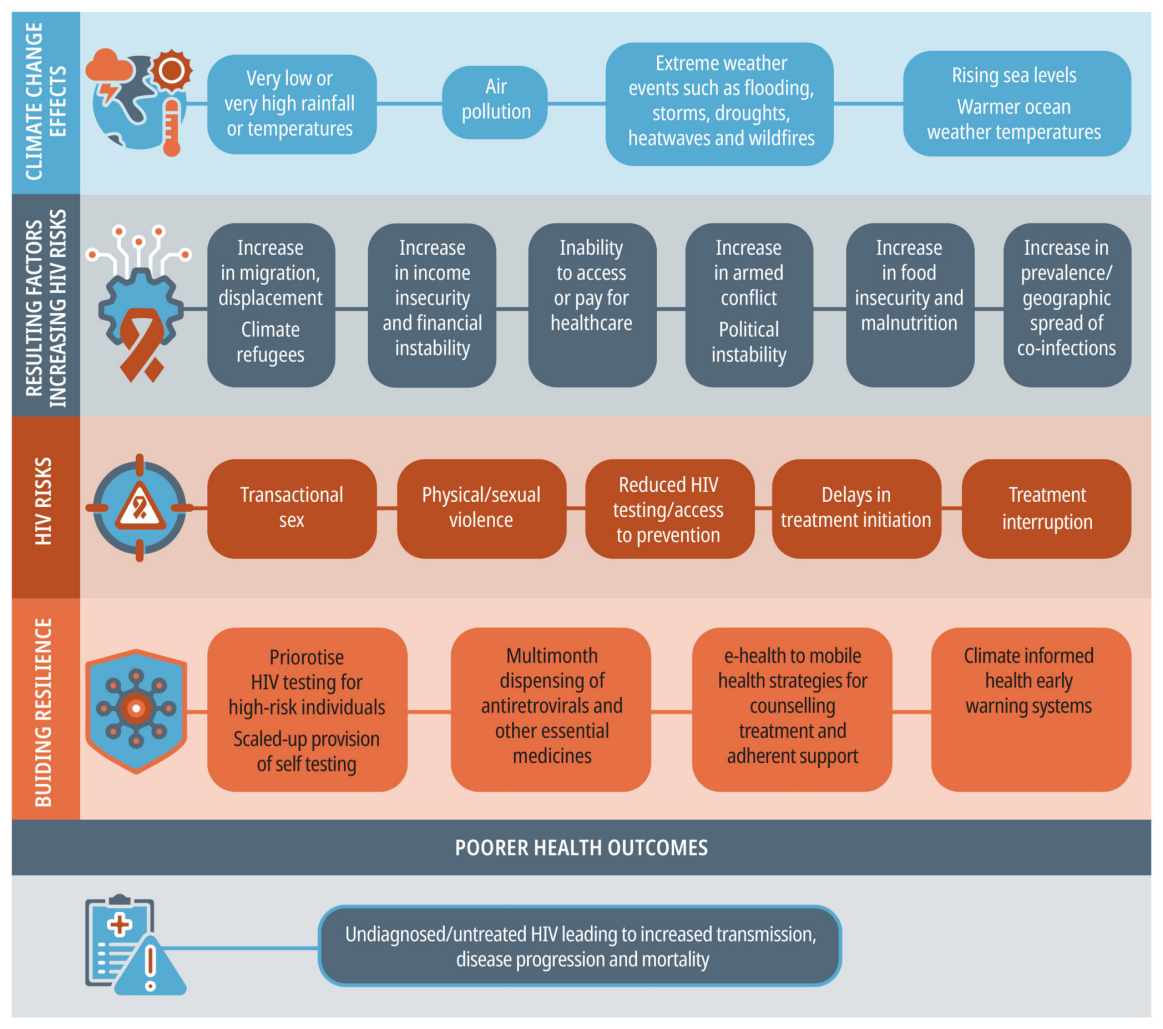
Conceptual framework of climate change impacts on HIV outcomes
